# Atlantic salmon (*Salmo salar* L.) post-smolts challenged two or nine weeks after seawater-transfer show differences in their susceptibility to salmonid alphavirus subtype 3 (SAV3)

**DOI:** 10.1186/s12985-016-0520-8

**Published:** 2016-04-11

**Authors:** J. Jarungsriapisit, L. J. Moore, G. L. Taranger, T. O. Nilsen, H. C. Morton, I. U. Fiksdal, S. Stefansson, P. G. Fjelldal, Ø. Evensen, S. Patel

**Affiliations:** Institute of Marine Research, Nordnesgaten 50, 5005 Bergen, Norway; Uni Research Environment, Uni Research, Thormøhlensgt., 49 B, 5006 Bergen, Norway; Department of Biology, University of Bergen, P.O. Box 7803, N-5020 Bergen, Norway; Institute of Marine Research, Matre Research Station, Matredal, Norway; Norwegian University of Life Sciences, Faculty of Veterinary Medicine and Biosciences, P.O. Box 8146 Dep, N-0033 Oslo, Norway

**Keywords:** Bath challenge, Bath immersion, Viral shedding, Salmon pancreas disease virus, SPDV, Pancreas disease, Plasma cortisol, ATPase activity, Condition factor

## Abstract

**Background:**

Pancreas disease (PD), caused by salmonid alphavirus (SAV), is an important disease affecting salmonid aquaculture. It has been speculated that Atlantic salmon post-smolts are more prone to infections in the first few weeks following seawater- transfer. After this period of seawater acclimatization, the post-smolts are more robust and better able to resist infection by pathogens. Here we describe how we established a bath immersion (BI) model for SAV subtype 3 (SAV3) in seawater. We also report how this challenge model was used to study the susceptibility of post-smolts to SAV3 infection in two groups of post-smolts two weeks or nine weeks after seawater - transfer.

**Methods:**

Post-smolts, two weeks (Phase-A) or nine weeks (Phase-B) after seawater- transfer, were infected with SAV3 by BI or intramuscular injection (IM) to evaluate their susceptibility to infection. A RT-qPCR assay targeting the non-structural protein (nsP1) gene was performed to detect SAV3-RNA in blood, heart tissue and electropositive-filtered tank-water. Histopathological changes were examined by light microscope, and the presence of SAV3 antigen in pancreas tissue was confirmed using immuno-histochemistry.

**Results:**

Virus shedding from the Phase-B fish injected with SAV3 (IM Phase-B) was markedly lower than that from IM Phase-A fish. A lower percentage of viraemia in Phase-B fish compared with Phase-A fish was also observed. Viral RNA in hearts from IM Phase-A fish was higher than in IM Phase-B fish at all sampling points (*p* < 0.05) and a similar trend was also seen in the BI groups. Necrosis of exocrine pancreatic cells was observed in all infected groups. Extensive histopathological changes were found in Phase-A fish whereas milder PD-related histopathological lesions were seen in Phase-B fish. The presence of SAV3 in pancreas tissue from all infected groups was also confirmed by immuno-histochemical staining.

**Conclusion:**

Our results suggest that post-smolts are more susceptible to SAV3 infection two weeks after seawater-transfer than nine weeks after transfer. In addition, the BI challenge model described here offers an alternative SAV3 infection model when better control of the time-of-infection is essential for studying basic immunological mechanisms and disease progression.

**Electronic supplementary material:**

The online version of this article (doi:10.1186/s12985-016-0520-8) contains supplementary material, which is available to authorized users.

## Background

Pancreas disease (PD) adversely affects the production of salmonids in many countries in Europe and North America, and is caused by an alphavirus commonly referred to as salmon pancreas disease virus (SPDV) or salmonid alphavirus (SAV). PD was initially recognized in Scotland in 1976 [1], and has been described in North America [2], Norway [3], Ireland [4], France, and Spain [5]. In Norway, PD outbreaks are mainly seen in the seawater phase of Atlantic salmon and rainbow trout aquaculture [6]. The alphavirus genome is composed of genes encoding non-structural proteins (nsP1-nsP4) for viral replication and structural proteins (E1-E3, capsid and 6K) [7]. Six subtypes of SAV (SAV1-SAV6) have been identified based on partial E2 and nsP3 genes [8]. Before 2011, only SAV3 had been associated with diagnosed PD-cases in Norway [6, 9]. In 2011, SAV subtype 2 (SAV2) was first detected in Norway [10] and its introduction was traced back to 2010 [11]. Since then, the number of SAV2 outbreaks has been increasing, although the severity of the disease associated with SAV2 is reportedly less severe than that caused by SAV3 [11, 12].

Smoltification, or parr-smolt transformation, is a complex biological process preparing salmonids for the seawater stage of their lifecycle. Smoltification involves profound biological changes to the endocrine, osmoregulatory, and immune systems resulting in alterations in morphology, physiology, and behaviour that prepares salmonids for life in the ocean [13]. During smoltification, growth hormone and cortisol act in synergy to improve the hypo-osmoregulatory ability of Atlantic salmon [14]. Gill Na^+^, K^+^-ATPase (NKA) activity is crucial to osmoregulatory adjustment in fish and it is used as an indicator of completion in smoltification [15]. Osmoregulatory system could be affected by viral infection [16]. After introduction to the marine environment, substantial changes in several components of the immune system have been observed: significant changes in the composition of leukocyte populations [17] and an increase in the concentration of total serum IgM [18] have been reported. Accordingly, it has been suggested that the initial period following seawater transfer is a time of high energy-demand, during which the fish are more susceptible to other stressors such as pathogens. After acclimatization to the marine environment is complete, post-smolts may be more robust and better able to handle stressors.

In order to gain a fundamental biological understanding of the immune response of salmon post-smolts to SAV3, a challenge model mimicking the infection in the field is vital. Intraperitoneal injection (*i.p*.) and intramuscular injection (*i.m.*) are commonly used in SAV challenge models [19–22]. The advantages of these methods are that the success of infection can, to some extent, be guaranteed, and time-of-infection and viral doses can be accurately controlled. However, neither *i.p*. nor *i.m.* injections represent the true natural route of infection, as the virus bypasses the host's first line of defense, such as skin and mucosal mucus. Cohabitation challenge has also been successfully used to mimic natural exposure to SAV [11, 23, 24]. However, the time-of-infection and the viral dose cannot be completely controlled when using a cohabitation model. Bath immersion (BI) is an alternative strategy that also mimics the natural exposure to water-borne virus. It was demonstrated that experimentally infected Atlantic salmon shed virus into the tank-water during the viraemic period [20]. Hence, the present study applied this knowledge to establish a BI challenge model for SAV3. BI can be a promising alternative model system to better study natural routes of infection, and gives the possibility to better control the viral dose and time-of-infection.

In the present study, we established a BI challenge model for SAV3 in seawater in order to investigate whether post-smolts were more susceptible to infection at either 2 weeks or 9 weeks after seawater-transfer. Here, we report virus shedding in water, viraemic period, viral load in the heart, and histopathological changes in the two groups of post-smolts following BI or IM challenge with SAV3 in seawater.

## Methods

### Fish and rearing conditions

The fish used in the present study were non-vaccinated Atlantic salmon post-smolts from the Aquagen strain, which had been produced as under-yearling smolts [25, 26] at the Institute of Marine Research (IMR) in Matre, Western Norway. The fish population was screened and confirmed to be negative for SAV3 and piscine reovirus (PRV). Two separate batches of fish from the same production cycle were transported to the rearing and disease challenge facility at IMR in Bergen. The first batch of freshwater smolts (average weight of 40.9 ± 6.5 g) was transferred into seawater upon arrival in Bergen (in early September 2013) and acclimatized in seawater for two weeks before infection experiments commenced. Thus, this first batch of experimental fish was challenged with SAV3 in the initial period after seawater transfer, and is hereafter referred to as Phase-A fish. The second batch of post-smolts (average weight of 88.8 ± 14.0 g) was transferred to Bergen (in October 2013) after being reared in seawater at Matre for seven weeks. Hence, this second batch of post-smolts was in a later period after seawater transfer and is hereafter referred to as Phase-B fish.

At IMR in Bergen, the fish from each batch were randomly distributed into 12 identical 250 L seawater tanks (12 °C, 34.5‰). Each tank contained 65 fish. The tanks were supplied with aeration (oxygen saturation of >85 %) and the water flow was maintained at 400 litres per hour (Lh^−1^). The fish were acclimatized in the experimental tanks for one week before SAV3 infection of shedders and for two weeks before SAV3 experimental challenges. The fish were fed twice daily to satiation with a commercial salmon feed (Spirit Supreme, Skretting, Norway). The fish were starved for 24 h prior to each sampling. The fish were bath anaesthetized with a mixture of metomidate (10 mg L^−1^) and benzocaine (60 mg L^−1^) before handling and injection, and euthanized with a mixture of metomidate (10 mg L^−1^) and benzocaine (160 mg L^−1^) before tissue sampling.

### Salmonid alphavirus (SAV)

Chum salmon heart-1 (CHH-1) cells were cultivated in 75 cm^2^ plastic cell culture flasks containing Leibovitz's L-15 medium (L-15) (Life Technologies, UK) supplemented with 10 % (v/v) foetal bovine serum (FBS) (PAA, France) at 20 °C. A SAV3 isolate from Atlantic salmon heart [27] was cultivated using CHH-1 cells in L-15 supplemented with 2 % FBS at 15 °C. The virus was harvested at 7 days post-infection (dpi) when the cytopathic effect (CPE) was observed. Quantification of virus stock was performed using the end-point dilution assay, and 50 % tissue culture infectious dose (TCID_50_) was calculated [28]. After this experiment was completed we discovered that the SAV3 isolate used was contaminated with low levels of infectious pancreatic necrosis virus (IPNV), as the isolate had earlier been passed through one round of infection in fish. However, IPNV contamination levels were relatively low (6 Ct values higher than SAV3). Anterior kidneys of IM Phase-A and BI Phase-A fish at 7, 14, 21 and 28 dpi were, therefore, analyzed for the presence of IPNV [29]. Twenty-five percent of the tested IM Phase-A fish were positive for relatively low levels of IPNV (average Ct value of 36), but all of the tested BI Phase-A fish were negative. Hence, co-infection with IPNV is not likely to have a major effect on the interpretation of the results of this study.

### Experimental design

**Fig. 1:**
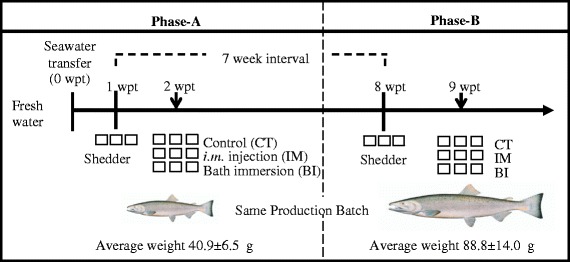
Experimental setup. Susceptibility to salmonid alphavirus subtype 3 (SAV3) of Atlantic salmon (*Salmo salar* L.) 2 weeks (Phase-A) or 9 weeks (Phase-B) post seawater- transfer (wpt). Triplicate tanks of shedder fish were intramuscularly (*i.m.*) injected with SAV3 approx. 1 week before the day of experimental challenges in order to obtain seawater containing SAV3 for BI challenge. Three treatments, control (CT), *i.m.* injection (IM) and bath immersion (BI) were performed in triplicate tanks. Arrows indicate the time of experimental SAV3 infection challenges

Both Phase-A and Phase-B experiments had a similar design, which is outlined in Fig. 1. One and eight weeks after seawater transfer of Phase-A and Phase-B fish, respectively, all the fish in 3 of the 12 tanks from each experiment were *i.m.* injected in the muscle in front of the dorsal fin and above the lateral line with 2 x 50 μl of SAV3. The titer of SAV3 in Phase-A and Phase-B was similar in the range of 2.0-3.7 x10^4^ TCID_50_ per fish. These fish served as shedder fish, whose purpose was to shed SAV3 into the tank-water. The water in these tanks (referred to as shedder tanks) was monitored for the presence of SAV3 by RT-qPCR. On the day of challenge, 7 days (in Phase-A) and 8 days (in Phase-B) after injection of shedder fish, three treatment groups in triplicate tanks were created: (*i*) fish *i.m.* injected with cell culture medium from uninfected CHH-1 cells, serving as non-infected control (referred to as ‘CT group’); (*ii*) fish *i.m.* injected with SAV3 (referred to as the ‘IM group’); (*iii*) fish infected by bath immersion in seawater (as described below) containing SAV3 (referred to as the ‘BI group’). The IM groups in Phase-A and Phase-B were given relatively similar titer of SAV3 (2.6–3.7x10^4^ TCID_50_ per fish). The experiments were carried out in strict accordance with guidelines and approved by Norwegian Animal Research Authority (NARA).

### Bath immersion (BI) challenge model

The BI groups were bathed in seawater from the shedder tanks as follows. The water flow in the 3 shedder tanks was stopped for one hour, and the tanks were supplied with extra aeration. After this 1 h period, the shedder fish were removed from the tanks and replaced by new fish. These BI fish were kept in the aerated shedder tanks with no water flow for six hours. The oxygen level in all tanks was closely monitored when the water flow was stopped. After 6 h of bath immersion, the water flow was resumed with normal aeration, which diluted out and removed the remaining virus in the tanks within a short time.

### Water sampling

Seawater sampling from the tanks, and up-concentration of SAV3 from the seawater samples, was performed [18] with some modifications. This water filtration technique is based on the VIRADEL (virus-adsorption-elution) method [30, 31] carried out by using electropositive, charge-modified, glass and cellulose (1MDS) filters [32, 33]. Briefly, one litre of seawater was collected from the tanks using sterile autoclaved screw-cap 1 L glass bottles. The water samples were vacuum filtered at a flow of 70–100 ml min^−1^ using a glass filtration system (MilliPore, USA.) through electropositive Zeta Plus™ 1MDS filters (Cuno Inc, USA.). After filtration, the filters were placed upside down in 55 mm petri-dishes containing 1 ml of lysis buffer (iPrep™ PureLink® Total RNA Kit, Invitrogen, USA.), and agitated at 500 rpm for 15 min on an orbital shaker. Four hundred microliters of the eluant was then transferred into a 1.5 ml tube and stored at -80 °C for RT-qPCR.

In the Phase-A experiment, water was collected from tanks containing IM fish at 2, 4, 6, 8, 10, 14, 18, 23 and 28 dpi, while water was collected from tanks containing the CT fish at 2, 4, 7, 10, 18 and 28 dpi. Water sampling in Phase-B was optimised based on the viral shedding profile seen in IM and CT groups in Phase-A. In the Phase-B experiment water was collected from tanks containing IM fish at 4, 8, 15 and 20 dpi, while water from tanks containing BI fish was collected at 1, 3, 5, 7, 10, 14 and 21dpi, and from tanks containing CT fish at 1, 8 and 15 dpi.

### Tissue sampling

Eight fish per tank per sampling point were euthanized as previously described, and length and weight were recorded before blood and tissue sampling. Blood was collected at 1, 3, 7, 10, 14, 21, and 28 dpi in Phase-A with heparinized syringes (for plasma) and Phase-B without anticoagulant (for serum) from the caudal vein. Plasma and sera were collected by centrifugation (9500 g, 10 min) within 3 h of sampling or after storage at 4 °C overnight, respectively. Gills from Phase-A fish were sampled and analyzed for Na^+^, K^+^-ATPase (NKA) activity at 1, 3, 7, 10, 14, 21 and 28 dpi by collecting gill filaments in 100 μl of ice-cold SEI buffer (150 mM sucrose, 10 mM EDTA, 50 mM imidazole, pH 7.3) and snap freezing in liquid nitrogen. Since Phase-B fish were expected to be stabilized and little influence of infection was expected, gills from these fish were not analysed for NKA activity. Hearts were collected at 3, 7, 14, 21, and 28 dpi for RT-qPCR analysis. Hearts from day 7, 14, 21 and 28 were cut in two longitudinally and the half that included the ventricle with bulbus arteriosus was snap frozen in liquid nitrogen while the other half including the atrium from four fish per tank was fixed in 10 % neutral buffered formalin. Plasma, sera, gill filaments and hearts were stored at -80 °C until further analyses. In addition, pancreatic tissue associated with pyloric caeca was sampled from four fish per tank at 7, 14, 21, and 28 dpi and fixed in 10 % neutral buffered formalin.

### RNA extraction and RT-qPCR

Total RNA was extracted from a homogenate of approx. 50 mg of heart tissue using an iPrep™ PureLink® Total RNA Kit (Invitrogen, USA.) with TRIzol® reagent (Ambion). The RNA isolation from filtered water eluants, plasma and serum samples was performed using the same kit with lysis buffer, according to the manufacturer’s instructions. The isolated RNA from plasma samples was lithium chloride precipitated to remove heparin which interfered with RT-qPCR. The AgPath-ID One-Step RT-qPCR Kit from Ambion, Life Technologies and a RT-qPCR assay targeting the SAV nsP1 gene were used for detection of SAV3 [34] with the following modification of the probe sequence FAM-5'-TCGAAGTGGTGGCCAG-MGB. The assay was performed with 200 ng of RNA (heart) or 2 μl of isolated RNA (plasma, sera and water), 400 nM of forward primer, 600 nM of reverse primer and 160 nM of probe in a total volume of 7 μl on a 384 well-plate. Amplification and fluorescence detection were measured by a 7900HT Fast Real-Time PCR system (Applied Biosystems) as recommended by the manufacturer. The threshold value for all samples was set to 0.1 and the amplification was run for 40 cycles. The elongation factor 1A (EFIA_A_) [35] was checked from a random selection of 25 % of heart samples from infected treatment groups representing all time-points of sampling to validate the quality of the RNA samples and all the samples showed stability and satisfactory levels of EFIA_A_ (Ct value of 21–22).

### Analysis of Na^+^,K ^+^ -ATPase (NKA) enzyme activity

In the Phase-A experiment, we analysed the NKA activity of gill filament samples from 12 fish per treatment at each time-point (1, 3, 7, 10, 14, 21, and 28 dpi) as representatives of each tank as described previously [15]. The NKA activity is expressed as μmol ADP mg protein^−1^ h^−1^.

### Analysis of plasma cortisol

On each sampling day, plasma samples from 12 individual fish in each treatment group from both Phase-A and Phase-B experiments were analyzed for cortisol concentration by ELISA according to the manufacturer’s recommendations (IBL International, Hamburg, Germany).

### Histology and immunohistochemistry (IHC)

The pancreas and heart tissues from all groups were fixed in 10 % neutral buffered formalin for 2 days before being processed and embedded in paraffin wax. Samples selected from fish with SAV3-positive hearts (identified using RT-qPCR) were sectioned at 3 μm using a Leica RM 2255 microtome (Leica Microsystems, Germany) and placed on SuperFrost® Plus slides (Menzel-Gläser, Germany). Selected histological sections (*n* = 3-9 from each group per sampling point of Phase-A and *n* = 2–4 from each group per sampling point of Phase-B) were stained with Haematoxylin-Erythrosin-Saffron (HES). The selected tissue sections were stained with an anti-E2 antibody to confirm the presence of SAV3. The paraffin sections were incubated at 60 °C for 30 min followed by de-waxing and dehydration with alcohol series and water. Antigen retrieval was carried out in 10 mM citrate buffer (pH 6.0) for 20 min followed by transferring to Tris-buffered saline (TBS, pH 7.4) using a 2100 Retriever model pressure cooker (Prestige Medical, England) according to the manufacturer’s instructions to unmask antigens. Blocking of non-specific antibody binding to sections was performed by using 5 % bovine serum albumin (BSA) in TBS for 20 min. All wash steps were carried out with TBS for 5 min until counterstaining. The primary antibody, a rabbit polyclonal anti-E2, was diluted 1: 200 in TBS containing 2.5 % BSA, and applied to the sections and incubated at 37 °C for 1 h, followed by biotinylated anti-mouse/rabbit immunoglobulin G, secondary antibody, and avidin-biotin alkaline phosphatase according to manufacturer’s recommendation (Vectastain universal ABC-AP kit; Vector Laboratories, California, USA). Sections were stained with the fuchsin substrate and chromogen system (Dako North America Inc., USA) and counterstained with Shandon’s haematoxylin. Positive immunostaining was visualised microscopically as a red/magenta colouration.

### Data and statistical analysis

Fulton’s condition factor was calculated by 100 × weight (g)/ [fork length (cm)]^3^. The Ct values of heart samples, gill NKA activities and plasma cortisol levels were imported into Statistica 12 (StatSoft, Inc., OK, USA). Differences in SAV3 RNA levels in the hearts of IM Phase-A and Phase-B fish were tested with a Mann-Whitney U non-parametric test, whereas One-way ANOVA was applied to NKA activity and plasma cortisol data.

## Results

### SAV3 challenges

Both Phase-A and Phase-B Atlantic salmon post-smolts were successfully infected with SAV3 in seawater via the *i.m.* injection route (IM group) and bath immersion route (BI group). No mortality was observed during the experimental period. SAV was not detected in the fish batch prior to the start of this experiment. However, 6 out of the 238 fish from the control group (CT) were subsequently found to be SAV-positive. Exposure to SAV during early life stages (before the transfer to Bergen and the start of the experiment) might have resulted in a very low prevalence of SAV, below the detection limit of our screening method, in our experimental fish population. Cross-contamination during fish/sample handling may also be a possible cause of this result.

### Condition factor

Fulton’s condition factors during the course of the experiment were in the range of 0.93–1.07 and 1.09–1.17 in Phase–A and Phase-B fish, respectively. The condition factor of IM Phase-A fish from 10 dpi onward was lower than the CT Phase-A and BI Phase-A fish with a significant difference at 28 dpi (*p* < 0.05, Additional file 1). No differences in condition factors were observed between groups in the Phase-B experiment.

### Gill NKA activity

Gill NKA activity of Phase-A post-smolts was 12–18 μmol ADP mg protein^−1^ h^−1^ (Additional file 2). The BI Phase-A fish showed significantly lower gill enzyme activity than those observed in the CT Phase-A fish at 28 dpi (*p* < 0.05; Additional file 2). Nevertheless, gill NKA activity levels were within normal range for post-smolts [36].

### Plasma cortisol

Plasma cortisol concentrations were relatively high in all groups (5.6–30.2 ng/ml in Phase-A, and 7.3–30.8 ng/ml in Phase–B fish, respectively). In Phase-A fish, the plasma cortisol concentrations in the CT group were significantly higher than the IM and BI groups at 1 dpi (*p* < 0.05) (Additional file 3). In the Phase-B experiment, plasma cortisol concentrations of BI Phase-B fish were significantly higher than CT Phase-B and IM Phase-B fish at 1 dpi (*p* < 0.05). BI Phase-B fish also showed significantly higher levels of plasma cortisol than IM Phase-B fish at 7 dpi (*p* < 0.05) (Additional file 3).

### Virus shedding

**Fig. 2:**
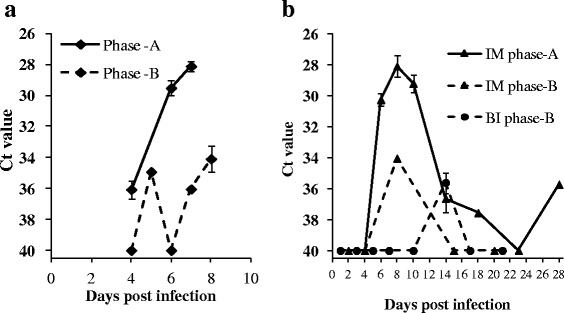
SAV3 detected in the tank-water by RT-qPCR. **a**, from tank containing shedder fish (♦) in Phase-A (solid line) and Phase-B (dashed line). Mean Ct value ± SEM; *n* = 1-3. **b**, from tanks containing the IM and BI groups. Viral shedding into the water from IM (▲) Phase-A (solid line), Phase-B (dashed line), and BI Phase-B (●) post-smolts at each time point. Mean Ct value ± SEM; *n* = 1-3. Ct value of 40 was set as negative

Prior to the start of the BI challenge, viral shedding from the shedder fish was monitored (Fig. 2a). In Phase-A, SAV3 could be detected in the tank water at the first sampling time point (4 dpi). Levels of SAV3 in the water were increased on day 6 and again on day 7. On day 7 the shedder fish were removed and the fish to be challenged were added to the tank as described. In Phase-B, the amount of virus was low or undetectable up until 6 dpi. By 8 dpi the level of virus had started to increase. In Phase-B, the shedder fish were removed from the tank at 8 dpi, and the challenge performed as described. Interestingly, Phase-A shedder fish shed significantly more SAV3 than the Phase-B shedder fish.

During the challenge experiments, virus shedding was monitored from CT Phase-A and -B fish, IM Phase-A and -B fish, and BI Phase-B fish (Fig. 2b). SAV3 was not detected in water samples from tanks containing the CT Phase-A and -B fish. SAV3 was detected in water from the IM Phase-A fish at 6 dpi, and peaked at 8 dpi. There was a considerable decrease in SAV3 levels from 10 dpi, and sporadic detection of SAV3 was observed at later time points until the end of the study at 28 dpi. In the Phase-B experiment, SAV3 shedding was detected from the IM Phase-B fish at 8 dpi (1 of 3 replicate tanks), and from the BI Phase-B fish at 14 dpi (2 out of 3 replicate tanks). Similar to the results from the shedder fish, the IM Phase-A fish shed higher levels of SAV3 than the IM Phase-B fish. Shedding from IM Phase-A fish also lasted for a longer time, than the shedding from IM Phase-B fish.

### Viraemia

SAV3 RNA was detected at 7 dpi in blood samples of all groups exposed to SAV3 (Table 1). Comparison of viral load in blood between Phase-A and Phase-B fish showed that the percentage of Phase-B fish with viraemia was lower than Phase-A (Table 1).

**Table 1:** Number of fish with viraemia

	IM	BI
Phase-A	16/18	7/15
	(89 %)	(47 %)
Phase-B	12/15	2/15
	(80 %)	(13 %)

Plasma (Phase-A) or serum (Phase-B) samples of IM and BI treatments at 7 dpi analyzed. The values represent the number of positive samples/number of analyzed samples, and percentage of positive samples (in brackets)

### SAV3 in the heart

**Fig. 3:**
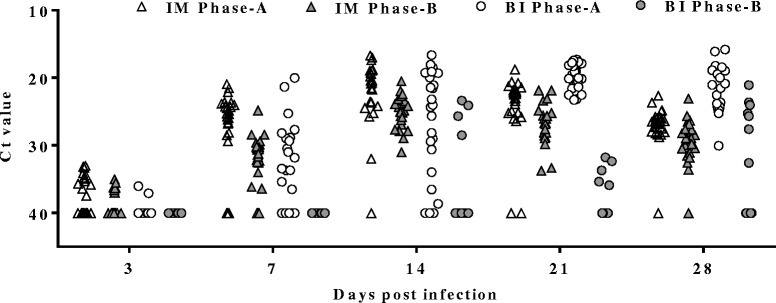
Viral load in heart**.** Ct value of individual fish from IM Phase-A (∆), IM Phase-B (▲), BI Phase-A (○) and BI Phase-B (●) at each time point; *n* = 24 except at: 14 dpi of BI Phase-A (*n* = 21), 7 dpi of IM Phase-B (*n* = 22), and 28 dpi of IM Phase-B (*n* = 23). Ct value of 40 was set as negative

**Fig. 4:**
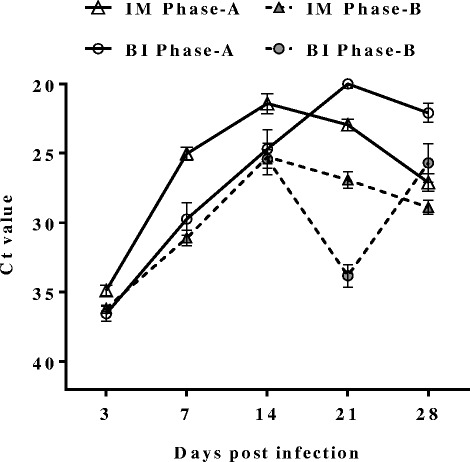
Mean Ct value of SAV-positive hearts. Ct values ± SEM from only the SAV3-positive hearts from IM Phase-A (∆), IM Phase-B (▲), BI Phase-A (○) and BI Phase-B (●) at each time point. *n* > 20 except at 3 dpi of IM Phase-A (*n* = 12); 7 dpi of BI Phase-A where *n* = 16; and 3 dpi of BI Phase-A and all time points of BI Phase-B where *n* = 2–7 (as very few fish were SAV3 positive)

**Fig. 5:**
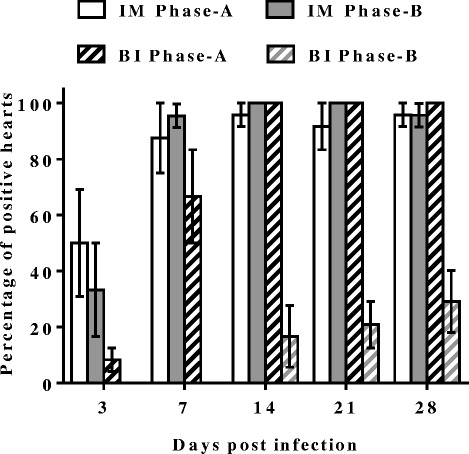
Prevalence of SAV-positive hearts. Bars represent mean percentage of positive samples ± SEM at each time point of IM Phase-A (white bar), IM Phase-B (grey bar), BI Phase-A (black diagonal stripe) and BI Phase-B (grey diagonal stripe)

SAV3 was detected in the hearts of IM and BI Phase-A fish, and in hearts from IM Phase-B fish at all sampling time-points while in hearts from BI Phase-B fish it was detected from 14 dpi (Fig. 3). The viral load in hearts from the IM Phase-A fish was significantly higher than that of IM Phase-B fish at all sampling time-points (*p* < 0.05) (Fig. 4). Interestingly, the viral load in the IM Phase-A fish, which peaked at 14 dpi, was lower than the viral load in the BI Phase-A fish at 21 dpi (Fig. 4). The percentage of positive fish was higher in IM Phase-A (50 %) than in IM Phase-B (33 %) at 3 dpi, and similarly high percentages of positive fish (88–100 %) were detected in both groups at later time-points (7, 14, 21 and 28 dpi) (Fig. 5). The percentage of positive individuals at 3 dpi was much lower in the BI Phase-A fish (8 %) than in the IM Phase-A fish (50 %), but it also reached nearly 100 % from 14 dpi (Fig. 5). There was a marked difference in the number of positive fish between BI Phase-A and BI Phase-B treatment groups (Fig. 5). In the BI Phase-B group, relatively few positive fish (17–29 %) were detected throughout the experiment (Fig. 5).

### Histopathology

**Fig. 6:**
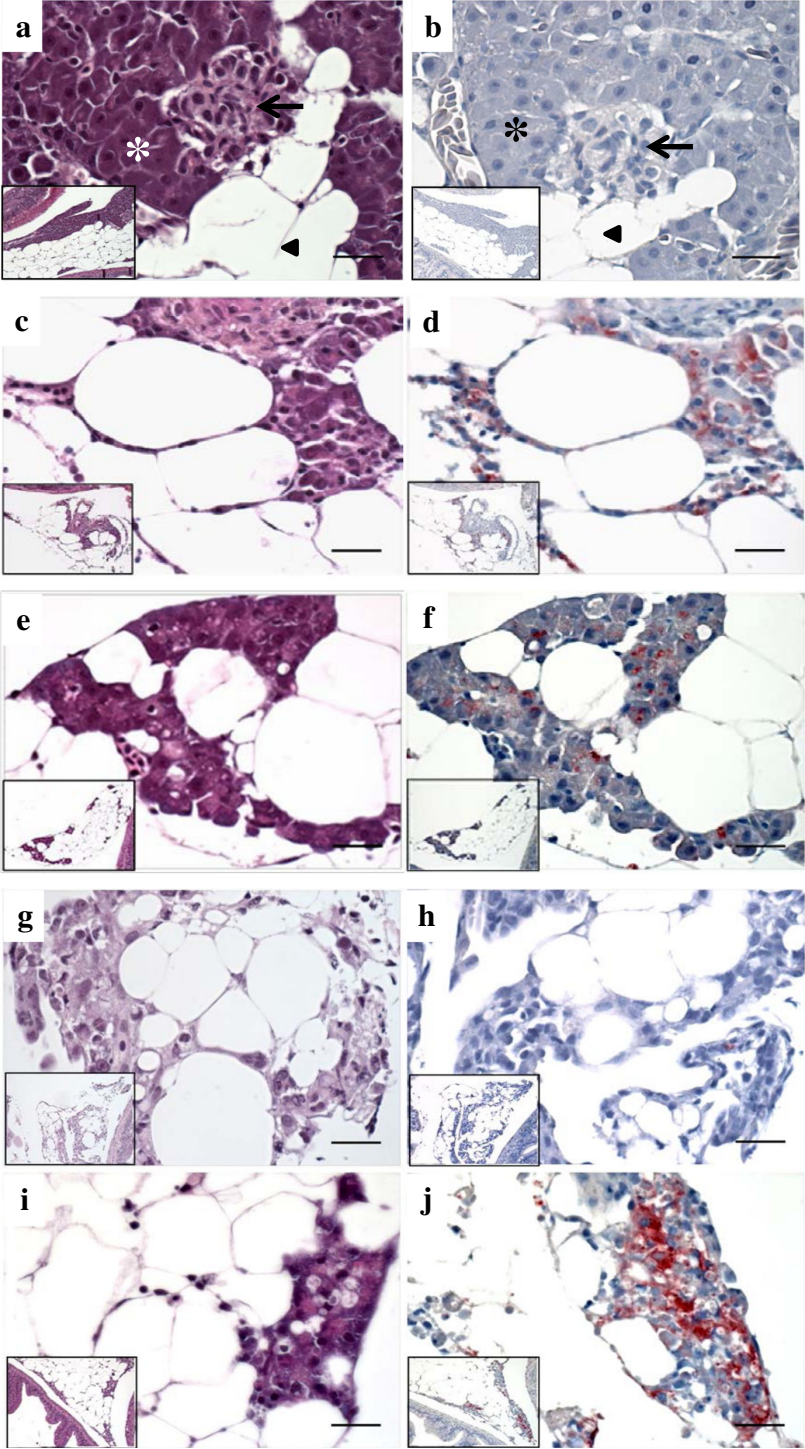
HES and IHC staining of pancreas from Phase-A post-smolts. **a** and **b**, CT group at 7 dpi; normal pancreatic tissue in Atlantic salmon with endocrine pancreatic tissue (arrow), exocrine pancreatic tissue with zymogen granules (*) and fat tissue (arrowhead) without red colour staining from IHC indicating the absence of SAV. **c** and **d**, IM group at 7 dpi; necrosis of exocrine pancreatic tissue with the presence of SAV confirmed by IHC. **e** and **f**, BI group at 7 dpi; normal appearance of pancreatic tissue, but the presence of SAV shown by IHC. **g** and **h**, IM group at 14 dpi; mononucleated cells (macrophage-like or fibroblast-like cells) replaced normal pancreatic tissue and weak staining for SAV by IHC. **i** and **j**, BI group at 14 dpi; necrosis of exocrine pancreatic tissue and strong staining for SAV by IHC. **a**, **c**, **e**, **g**, and **i**, HES staining. **b**, **d**, **f**, **h**, and **j**, IHC staining. Bar 50 μm. The insets were at 200x

**Fig. 7:**
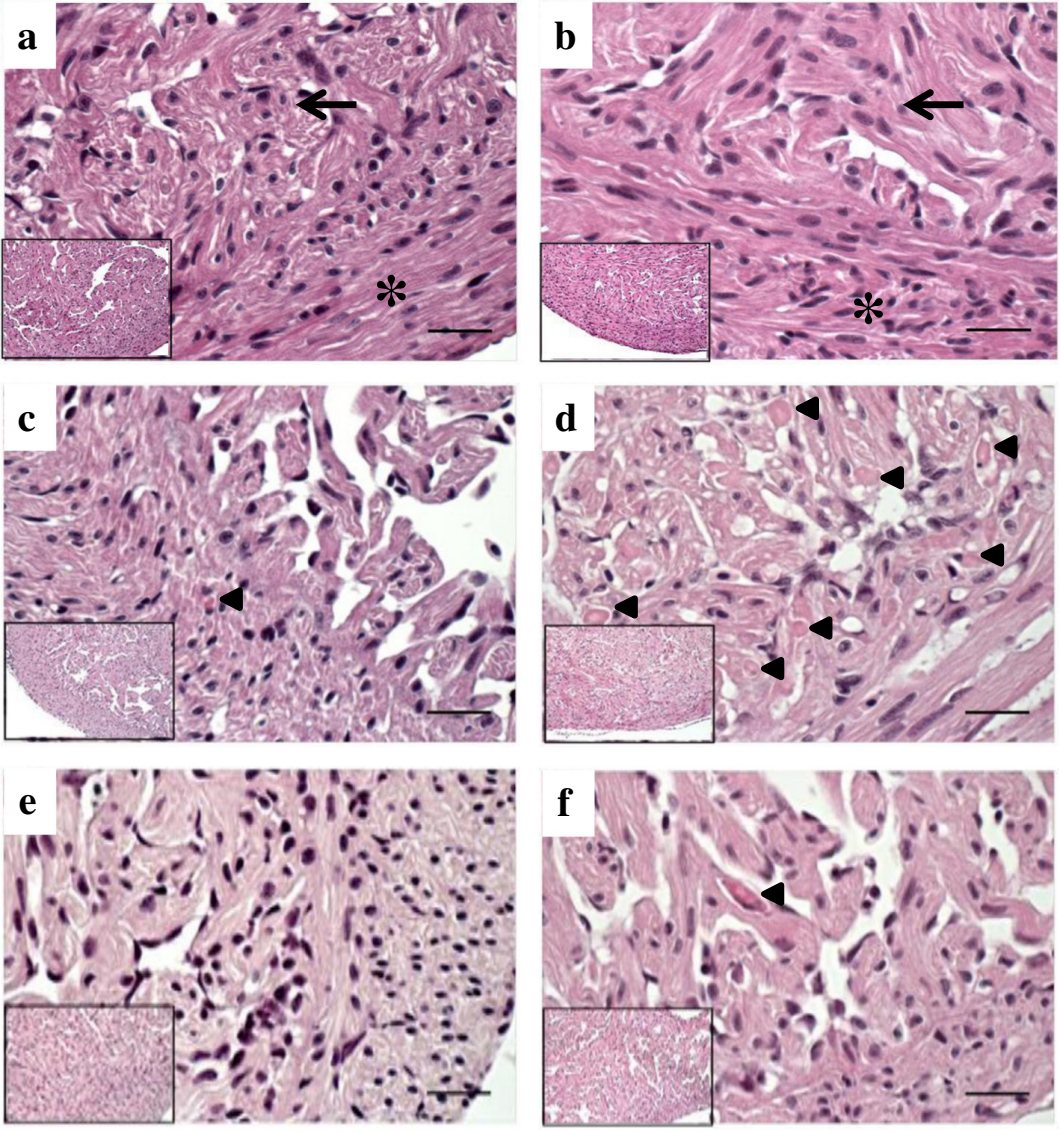
HES staining of heart from Phase-A post-smolts at 7 dpi and 14 dpi. **a** and **b**, CT group; normal heart tissue in Atlantic salmon showing cardiomyocytic cells in compact (*) and spongy (arrow) layers. **c** and **d**, IM group; focal (**c**) and severe diffused (**d**) myocardial degeneration. **e** and **f**, BI group; normal appearance of heart tissue (**e**) and focal myocardial degeneration (**f**).**a**, **c**, and **e**, 7 dpi; **b**, **d**, and **f**, 14 dpi**.** Arrowhead shows necrosis of myocardiocytes. Bars 50 μm. The insets were at 200x

**Fig. 8:**
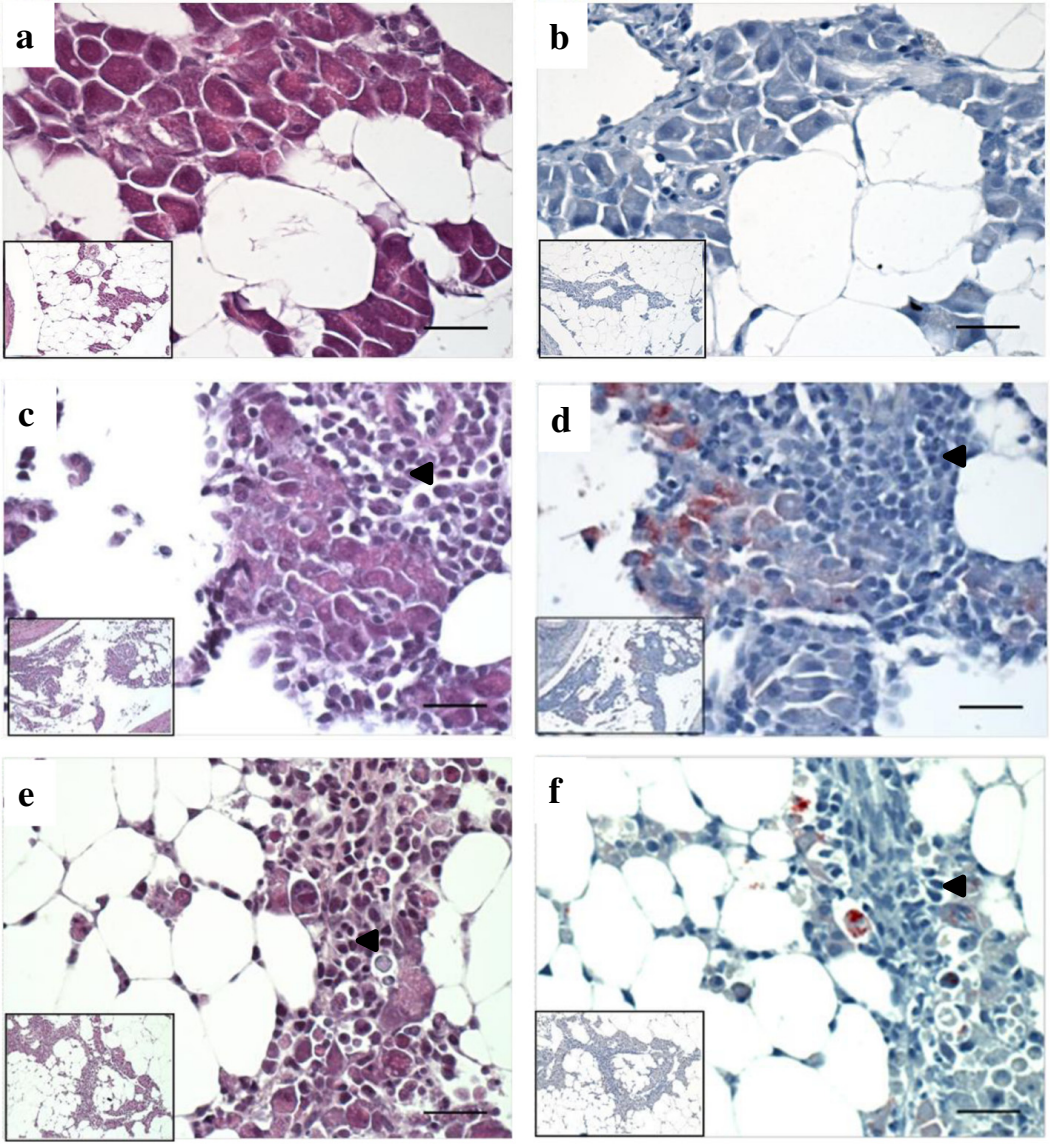
HES and IHC staining of pancreas from Phase-B post-smolts. **a** and **b**, control group at 14 dpi; normal pancreatic tissue in Atlantic salmon with exocrine pancreatic tissue with zymogen granule and fat tissue without red colour staining from IHC indicating the absence of SAV. **c** and **d**, IM group at 7 dpi; necrosis of exocrine pancreatic tissue with the presence of SAV confirmed by IHC. **e** and **f**, BI group at 7 dpi; focal pancreatic acinar cell necrosis with the presence of SAV shown by IHC. **a**, **c**, and **e**, HES staining; **b**, **d**, and **f**, IHC staining. Arrowhead shows infiltration of inflammatory cells. Bars 50 μm. The insets were at 200x

Histopathological changes in pancreas and heart tissue of SAV3-positive fish from all SAV3-exposed groups was examined, and the presence of SAV3 was confirmed by immunohistochemistry (IHC). Extensive necrosis of exocrine pancreatic cells, and degeneration of myocardial cells in the heart, was observed in IM Phase-A and BI Phase-A fish with differences in time of onset (Figs. 6 and 7). At 7 dpi, necrosis of exocrine pancreatic tissue and detection of SAV3 was observed in the IM Phase-A fish (Fig. 6c-d), whereas pancreatic tissue in the BI Phase-A fish showed a relatively normal histological appearance regardless of the presence of SAV3 in pancreatic tissue (Fig. 6e-f). Overall, a similar degree of severity of PD-associated histopathological changes in the pancreas and heart from IM Phase-A fish was also seen in the BI Phase-A fish approximately one week later. Significant necrosis of exocrine pancreatic tissue occurred at 7 dpi in the IM Phase-A fish (Fig. 6c-d) and at 14 dpi in the BI Phase-A fish (Fig. 6i-j). Focal myocardial degeneration was seen at 7 dpi in the IM Phase-A fish (Fig. 7c) and at 14 dpi in the BI Phase-A fish (Fig. 7f). Severe diffuse myocardial degeneration was observed at 14 dpi in the IM Phase-A fish (Fig. 7d). Both IM and BI Phase-B fish showed mild histopathological changes in the pancreas and infiltration of inflammatory cells (Fig. 8c-f) could be seen when compared to Phase-A fish (Fig. 6c-j).

## Discussion

Here, we report that a BI model for SAV3 infection of Atlantic salmon in seawater was successfully established. To the best of our knowledge, this is the first report of a SAV3 BI challenge model for Atlantic salmon post-smolts in seawater. While injection (*i.m.* or *i.p.*) challenge models for SAV3 (and many other viruses) provide control of viral dose and time-of-infection, they do not represent the true natural route of infection. On the other hand, cohabitation challenge models mimic natural route of exposure, but control of viral dose and time-of-exposure is inferior to the injection challenge model. Importantly, the bath immersion model established in the present study also allows natural exposure of challenged fish to SAV3. In contrast to cohabitation challenge, our bath immersion model gives a more accurate estimation of time-of- exposure by limiting the time-window to 6 h. Furthermore, an additional advantage over co-habitation models is that our BI model gives better control of the viral dose that the fish are actually exposed to during the 6-h window compared to cohabitation challenge model where exposure occurs over the period of viral shedding, possibly several days.

In this study, two groups of Atlantic salmon from the same production batch were challenged with SAV3, by *i.m.* injection or by bath immersion, 2 weeks (Phase- A) or 9 weeks (Phase- B) after seawater transfer (Fig. 1). Injection of SAV3 showed that there were differences in viral shedding, viral load in the heart, and degree of histopathological changes in various tissues between Phase-A and -B fish. Unsurprisingly, since all fish were injected with virus, the prevalence of SAV3 positive fish in the Phase-A and -B groups was rather similar. However, when Phase-A and -B fish were challenged with SAV3 using the newly established BI challenge model (that mimics the natural exposure to water-borne virus), variation in all of the parameters mentioned above, including the number of SAV3 positive fish, was seen.

In the BI model the exposure dose relies on the amount of SAV3 shed from the shedder fish. Our results show that shedder fish from Phase-A, which were injected with SAV3 1 week after seawater-transfer shed more virus than the shedders from Phase-B, which were injected 8 weeks after seawater transfer (average Ct value in water of 28 and 34, respectively, Fig. 2a). Thus, the BI fish in Phase-A were exposed to a greater amount of virus than the BI fish in Phase-B. This may explain the differences observed between Phase-A and -B fish using the BI challenge model. Our data therefore suggest that using small post-smolts in the first weeks after seawater transfer as shedder fish is an optimal solution for obtaining maximum and most consistent virus shedding.

We also note that a “cohabitation effect” may also have occurred in the Phase-B BI challenge since there was a mixture of infected and possibly naive individuals in the population throughout the experimental period. Therefore, the length of the experiment would have theoretically allowed a second exposure to SAV3 shed from the few fish that were infected in the first round of exposure. This likely reflects how the virus is transmitted in the field. If required, it may be possible to prevent this second round of exposure in the BI model by optimizing the initial infection dose or by limiting the duration of the study. However, studying the viral shedding profile of fish infected with SAV3 via BI may also be very important for understanding virus transmission from fish-to-fish in a field situation. Such studies will be useful for developing management strategies during PD outbreaks. Thus, the BI model described here may contribute to a better understanding of field outbreaks of viral diseases and provide a greater insight into salmon immune responses to SAV3.

Virus shedding is an indicator of viral infection and multiplication in the host. Previously, it has been demonstrated that SAV3 is shed into the water through mucus and faeces [37], and that there is a positive correlation between viraemia and virus shedding [20, 23]. Our results showed that IM fish in Phase-A shed more virus than IM fish in Phase-B. This suggests that SAV3 multiplication was more efficient in Phase-A fish and, therefore, more virus particles were shed. It is worth noting that detection of SAV3 from water samples using VIRADEL method followed by RT-qPCR has its limitations. In the Phase-A study we observed sporadic detection of the virus in the water (after the peak period). The limitations in the sample collection, sample processing and/or analysis can be overcome by further optimization.

More fish with viraemia could be detected in the IM and BI Phase-A fish as compared to IM and BI Phase-B fish. SAV3 in blood or in tank-water could be detected for longer period (up to 28 dpi) in Phase-A post-smolts when the fish were small (41 g). This is similar to previous results with 40 g salmon parr (up to 105 dpi) [21]. It appears to be shorter (up to 14 dpi) in bigger post-smolts (89 g) at later period after seawater transfer (Phase-B in the present study), as it was also seen in other published results for 73 g post-smolt (4–13 dpi) [20], and 120 g (3–14 dpi) [38]. The longer shedding period in Phase-A post-smolts is likely due, at least in part, to a reduced capacity to clear the virus in these fish compared to Phase-B post-smolts. Possibly virus replication in the muscle tissue [39] has also contributed to the detection of virus in the water sample at 28 dpi. SAV3 was detected in tank-water and sera from Phase-B BI fish indicating that the virus was successfully transmitted by the BI model. Since shedding is a reflection of viraemia, this suggests that shedding of SAV3 by the BI fish may have occurred at 7 dpi, but that it was most likely below the detection limit. It is also possible that the peak of viraemia in the BI fish might occur later than 7 dpi, since SAV3 was detected in the tank-water from BI Phase-B fish at 14 dpi.

Quantification of SAV3 nsP1-RNA revealed significant differences in the viral load in the heart between Phase-A and -B fish, which may indicate a difference in immune response to SAV3 between these two stages. In the present study, factors affecting the difference in immune response could be fish size and/or period after seawater transfer. Since we used fish from the same production batch, differences due to genetic variation should be minimal. The BI Phase-A fish showed a higher peak of viral load in the heart than the IM Phase-A fish, but with a one week time-lag (21 versus 14 dpi). The route of uptake may play a role here: SAV3 infection via the water-borne exposure may lead to a greater level of infection, even though the virus takes a longer time to multiply and peak, due to the lower number of virus particles responsible for the initial infection. In supporting of this theory, it has been previously reported that waterborne infection (cohabitation) produced a greater severity of PD than *i.p.* injection [11]. The percentage of positive individuals was similar in IM Phase-A and -B, whereas substantial differences were seen between the BI groups from both phases. The percentage of SAV3 positive fish in IM Phase-A and -B were similar because injection introduced virus directly into the host body.

The histopathology seen in experimentally infected post-smolts was typical of clinical PD [38, 40]; necrosis and loss of exocrine pancreatic tissue and degeneration of cardiomyocytes, where pancreatic necrosis was observed before heart lesions. Phase-A post-smolts in both IM and BI groups showed more extensive histopathological changes compared to Phase-B post-smolts reflecting the difference in susceptibility to SAV3 between these groups. The positive correlation between viral load and histopathological changes observed in the present study is in agreement with an earlier report [39]. A strong correlation between viral load and severity of histopathology in heart was very clear amongst Phase-A post-smolts, with increasing viral load coinciding with more severe PD lesions. Phase-B post-smolts appeared to show a similar trend, but a firm conclusion was difficult to draw due to SAV3-positive fish from Phase-B showing only mild PD lesions in heart. The presence of SAV3 in pancreas was confirmed by IHC, and Phase-A post-smolts displayed more prominent staining in the pancreas and more severe PD-related lesions. The most pronounced SAV3 staining could be seen in the exocrine pancreatic cells showing necrosis similar to a previous study [40]. Interestingly, SAV3 staining was also observed in some exocrine pancreatic cells of BI Phase-A fish, which were otherwise of normal appearance, suggesting a very early stage of infection with a low viral load in the tissues. In addition, infiltration of inflammatory cells could be seen as early as 7 dpi in Phase-B post-smolts indicating activation of a cellular immune response to SAV3.

Taken together the results presented here strongly suggest that: (i) Atlantic salmon post-smolts are highly susceptible to SAV3 infection 2 weeks after seawater transfer (Phase A fish); and (ii) post-smolts challenged with SAV3 9 weeks after seawater transfer (Phase B fish) are less susceptible to SAV3 infection. Thus the Phase-A post-smolts were infected with SAV3 during their initial acclimatization in seawater when their ability to mount an anti-viral immune response was inferior to Phase-B post-smolts. In previous studies, differences in susceptibility to SAV3 and other fish viruses have been attributed to fish strains [38], age and size [41, 42], or environmental conditions [43]. In this study genetic variation was minimized by using fish from the same production batch. Therefore, factors affecting susceptibility between these two post-smolt groups might be age, size, and/or physiological or immunological status of the fish after sea water transfer. A combination of age and size has been shown to affect the virulence of infectious hematopoietic necrosis virus (IHNV) resulting in differences in mortality [41]. Smoltification is a developmental event involving endocrine, morphological, physiological and behavioural aspects that allow salmonids to enter the ocean [13]. After seawater-transfer, marked changes in the immune system have been reported [17, 18]. Directly after seawater-transfer, post-smolts (Phase-A fish) are utilizing large amounts of energy acclimatizing to the marine environment, and may be less able to mount an effective immune response. Successful adaptation to the seawater occurs after a few weeks [44], when post-smolts are more robust and better able to fight a SAV3 infection (as seen for Phase B fish). Gill NKA activity from our study was within the normal range so, presumably, no secondary physiological disturbances occurred. The IM Phase-A fish showed a significant decrease in Futon’s condition factor at the end of the experiment indicating that SAV3 infection contributes to weight loss, and this has also been shown in previous studies [11, 45]. The statistically significant differences in concentrations of plasma cortisol may have resulted from a combination of the handling stress, injections, and viral infection. However, the concentration of plasma cortisol was similar to previous reports [44, 46]. A specific trend or substantial effect of stress, due to disease progression, was not evident in this study and future studies may benefit from measuring several components of the stress-axis, since turnover of cortisol may vary with hormone production, receptor abundance, and clearance of the ligand.

## Conclusions

In the present study, a BI challenge model for SAV3 in seawater was successfully established. Using the IM and BI challenge models we demonstrated that post-smolts challenged with SAV3 2 weeks after seawater-transfer are more susceptible to infection than post-smolts challenged 9 weeks after seawater-transfer.

## Additional files

Additional file 1: Condition factor of Atlantic salmon post-smolt in Phase-A experiment. The additional file condition factor.ppt shows Mean ± SEM from CT (■), IM (▲) and BI (●) groups at each time point (days post infection), *n* = 24. (PPT 73 kb) Additional file 2: Gill NKA activity of Atlantic salmon post-smolt in Phase-A experiment. The additional file gill NKA activity. ppt shows mean ± SEM from CT (■), IM (▲) and BI (●) groups at each time point (days post infection). *n* = 12, except control group at 21 dpi, *n* = 11. (PPT 75 kb) Additional file 3: Concentration of plasma cortisol. The additional file plasma cortisol.ppt shows mean ± SEM from CT (open bar), IM (grey bar) and BI (diagonal stripe) groups in Phase-A (A) and Phase-B (B) at each time point (days post infection), *n* = 11–12. (PPT 82 kb)

## Abbreviations

BI: bath immersion
CT: control
Ct value: cycle threshold value
dpi: days post infection
IM: intramuscular injection
*i.m.*: intramuscular
*i.p.*: intraperitoneal
PD: pancreas disease
Phase-A: two weeks after seawater- transfer
Phase-B: nine weeks after seawater- transfer
RNA: ribonucleic acid
RT-qPCR: quantitative reverse transcription polymerase chain reaction
SAV: salmonid alphavirus
SPDV: salmon pancreas disease virus
wpt: weeks post seawater- transfer
