# Supplementary figures and images for: Atlantic salmon (Salmo salar L.) post-smolts challenged two or nine weeks after seawater-transfer show differences in their susceptibility to salmonid alphavirus subtype 3 (SAV3)
Source: Virol J. 2016 Apr 11;13:66. doi: 10.1186/s12985-016-0520-8 (PMC4827186; doi:10.1186/s12985-016-0520-8)

## Slide 1
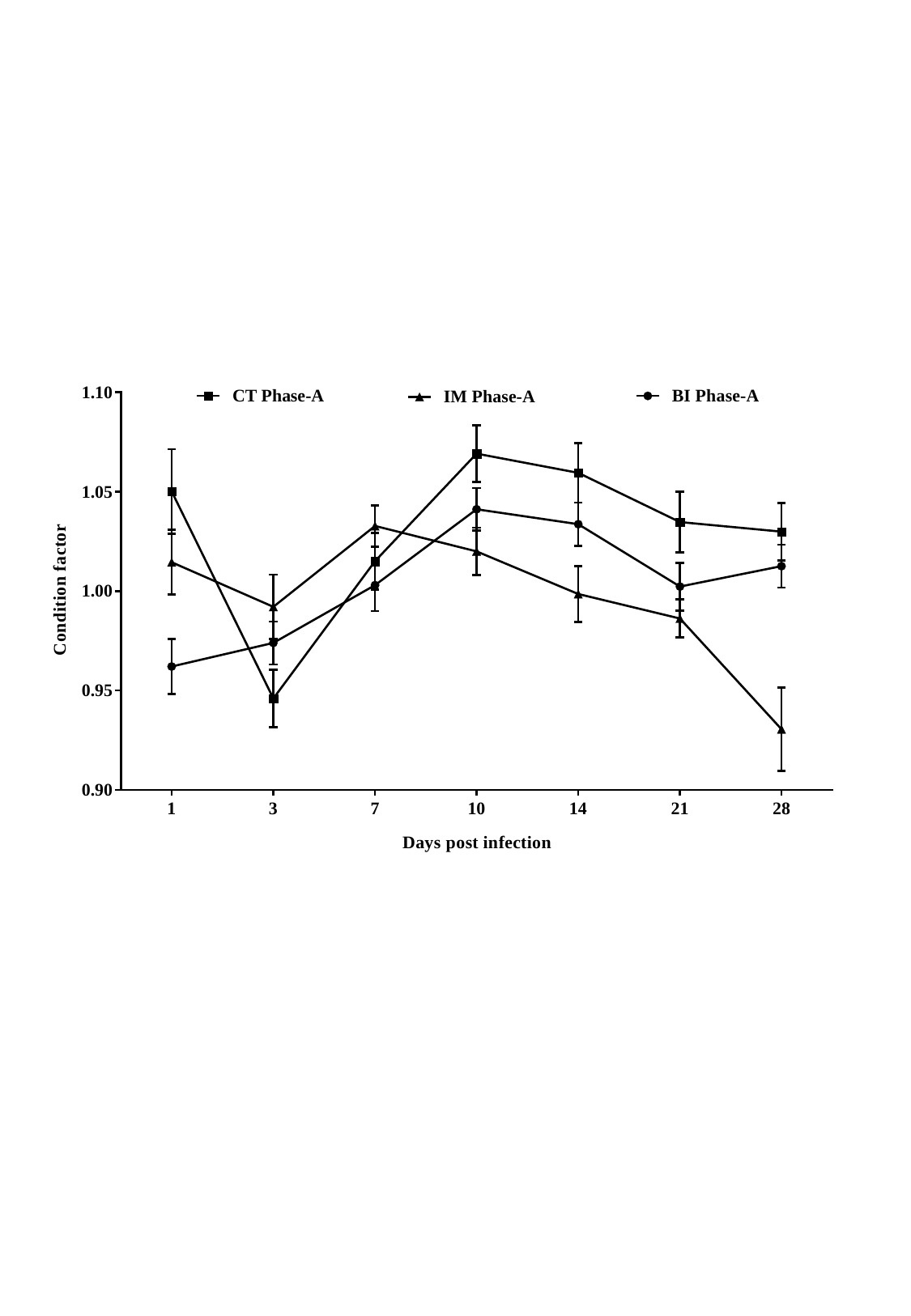

Supplement: Additional file 1: — Condition factor of Atlantic salmon post-smolt in Phase-A experiment. The additional file condition factor.ppt shows Mean ± SEM from CT (■), IM (▲) and BI (●) groups at each time point (days post infection), n = 24. (PPT 73 kb) [file 12985_2016_520_MOESM1_ESM.ppt]

## Slide 1
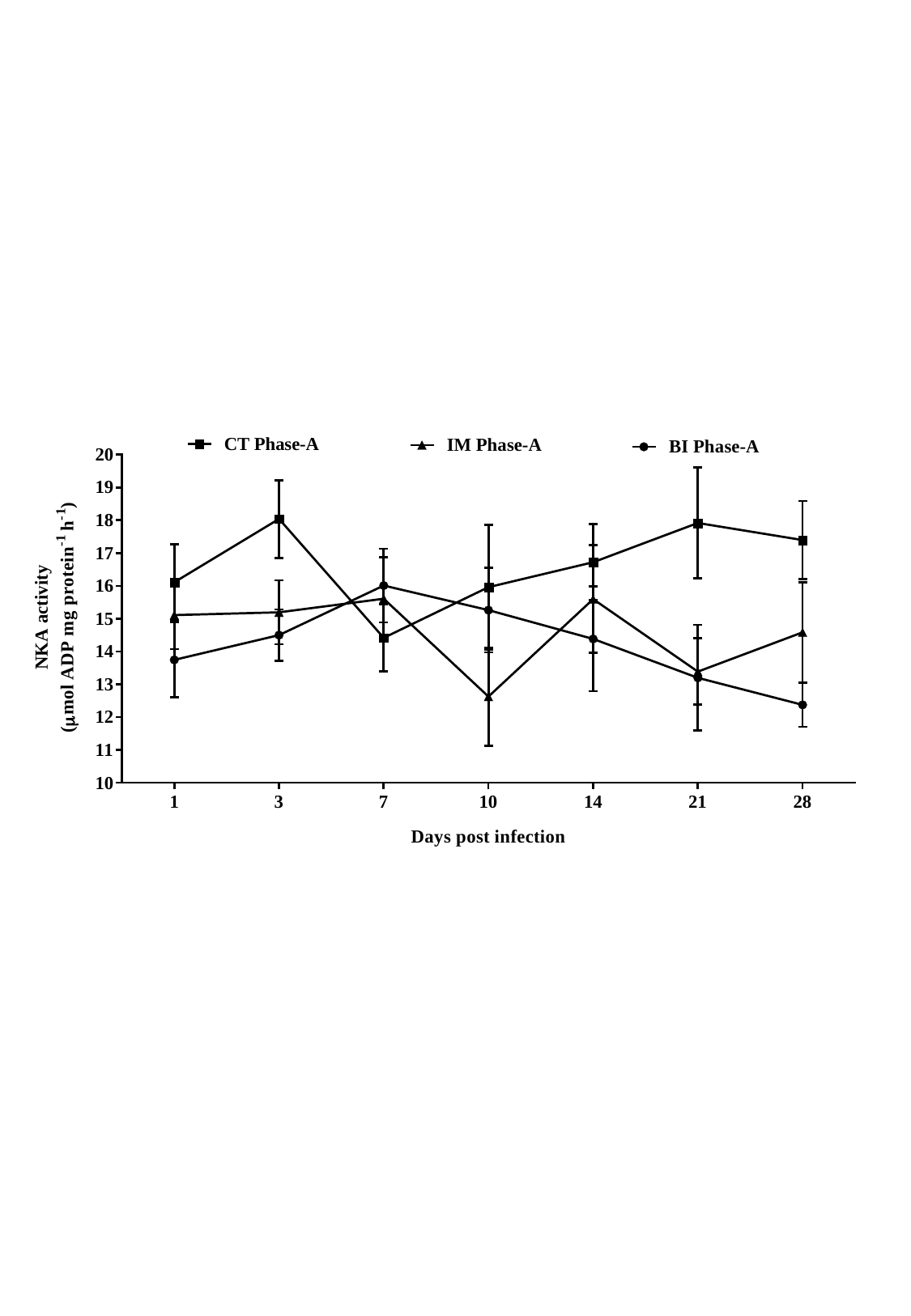

Supplement: Additional file 2: — Gill NKA activity of Atlantic salmon post-smolt in Phase-A experiment. The additional file gill NKA activity. ppt shows mean ± SEM from CT (■), IM (▲) and BI (●) groups at each time point (days post infection). n = 12, except control group at 21 dpi, n = 11. (PPT 75 kb) [file 12985_2016_520_MOESM2_ESM.ppt]

## Slide 1
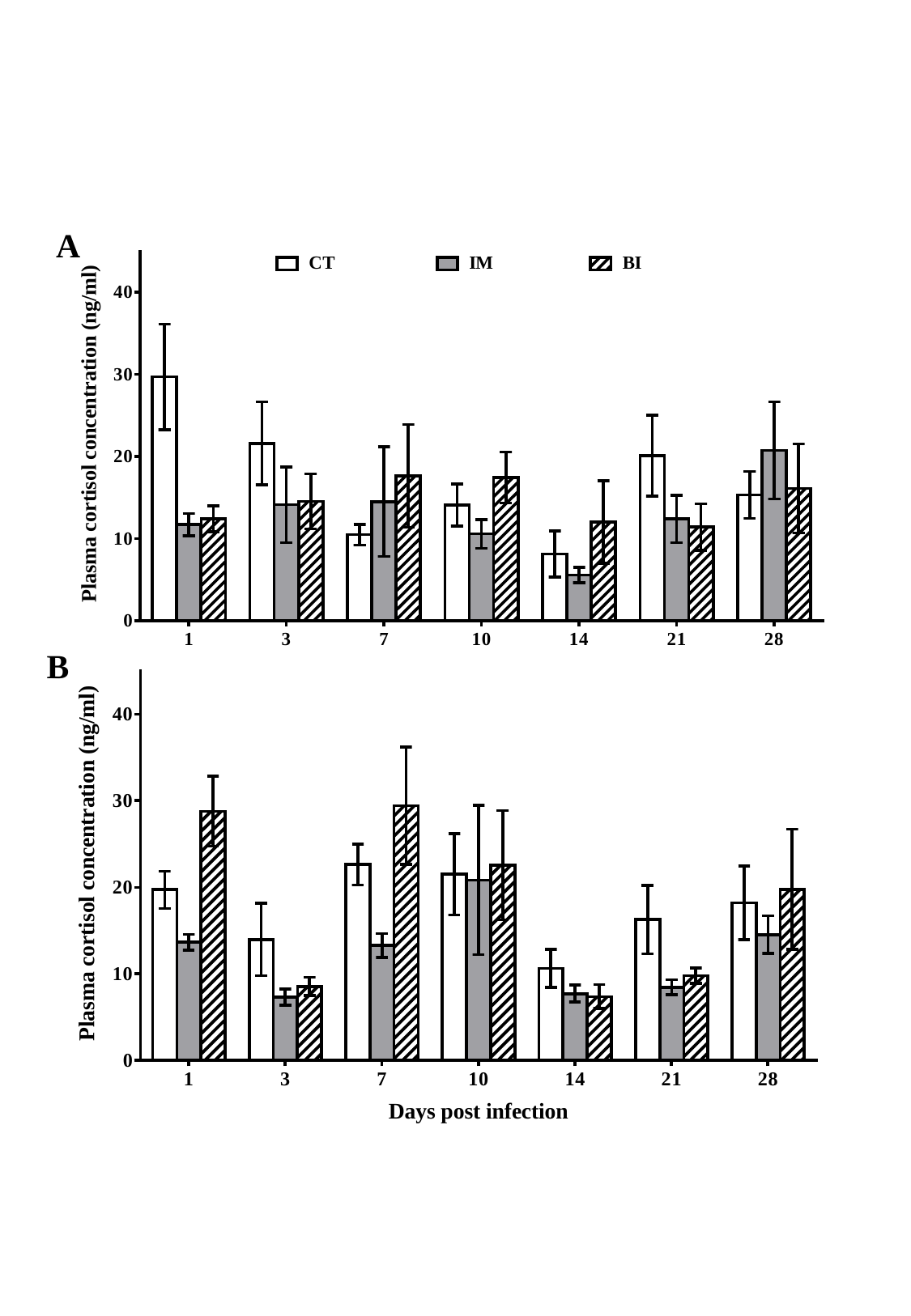

A
B

Supplement: Additional file 3: — Concentration of plasma cortisol. The additional file plasma cortisol.ppt shows mean ± SEM from CT (open bar), IM (grey bar) and BI (diagonal stripe) groups in Phase-A (A) and Phase-B (B) at each time point (days post infection), n = 11–12. (PPT 82 kb) [file 12985_2016_520_MOESM3_ESM.ppt]
